# Children’s spontaneous emotional expressions while receiving (un)wanted prizes in the presence of peers

**DOI:** 10.3389/fpsyg.2015.01401

**Published:** 2015-09-17

**Authors:** Mandy Visser, Emiel Krahmer, Marc Swerts

**Affiliations:** Tilburg Center for Cognition and Communication, Tilburg University, TilburgNetherlands

**Keywords:** emotional expressions, contextual factors, social presence, development, (re)appraisals, mistaken-gift-paradigm, dissappointment

## Abstract

Although current emotion theories emphasize the importance of contextual factors for emotional expressive behavior, developmental studies that examine such factors are currently thin on the ground. In this research, we studied the course of emotional expressions of 8- and 11-year-old children after winning a (large) first prize or a (substantially smaller) consolation prize, while playing a game competing against the computer or a physically co-present peer. We analyzed their emotional reactions by conducting two perception tests in which participants rated children’s level of happiness. Results showed that co-presence positively affected children’s happiness only when receiving the first prize. Moreover, for children who were in the presence of a peer, we found that eye contact affected children’s expressions of happiness, but that the effect was different for different age groups: 8-year-old children were negatively affected, and 11-year-old children positively. Overall, we can conclude that as children grow older and their social awareness increases, the presence of a peer affects their non-verbal expressions, regardless of their appreciation of their prize.

## Introduction

In December 2011, an enormous hit on YouTube followed when an American talk show host, Jimmy Kimmel, asked members of his audience to film their kids when they were given a Christmas present their parents were sure they would not like (Jimmy Kimmel Live! ABC 2011). While unwrapping their brand new onion or deodorant stick, most children screamed, got rather upset and eventually threw the unwanted gift away. However, when they were in the company of a sibling, the children’s reactions tended to alter considerably, in that, depending on the context, the presence of the other child occasionally seemed to increase the level of frustration, or, interestingly enough, turn the child’s initial disappointment into a more positive feeling. This was especially the case when their brother or sister was given a present that the child would judge as a (slightly) better or worse alternative. In of one of the Jimmy Kimmel video clips^1^, a boy appeared to be relatively excited about the Christmas present he received, a well-sized potato, as he seemed to judge this as a better gift than his older brother’s, who got paper letters spelling “3DS” (which is the name of a then popular game console). While his younger brother appeared to get more and more content with his gift, the older boy seemed to become more distressed with his own. Perhaps, observing the enjoyment of his younger brother was important for the boy’s evaluation of his own gift.

This example demonstrates that the presence of a peer may urge children to express their feelings more intensely, in either a positive or negative direction. It is likely that if both siblings in the Jimmy Kimmel fragment had been alone while unpacking their gifts, their emotional expressions would have been different, since they would not have to take each other’s disappointment or enjoyment into account for the evaluation of their own present. Indeed, a review of existing theories of emotion reveals that researchers have claimed that external factors like social context may affect the way emotions are expressed (e.g., Frijda, 1986; Russell and Feldman Barrett, 1999; Scherer et al., 2001; Scherer, 2009; Mumenthaler and Sander, 2012). However, to our knowledge, so far no studies have examined how these context-dependent emotion theories apply to the way other people’s responses affect children’s emotional expressions. In this study, we concentrate on three factors that may influence children’s non-verbal expressions.

The first factor we consider is the presence or absence of a peer, where we examine whether this influences how children display different emotional expressions in response to disappointing or satisfying presents. In general, children may be expected to react politely (e.g., by smiling) when they receive a present, regardless of whether they appreciate it or not (e.g., Kieras et al., 2005). Earlier studies on this topic focused on factors like age (Saarni, 1984; Cole, 1986; Garner and Power, 1986; Kieras et al., 2005; Kromm et al., 2015), culture (Garrett-Peters and Fox, 2007), the presence of parents (Zeman and Garber, 1996) and particular response strategies children may use when receiving a disappointing gift (Baaken, 2005; Tobin and Graziano, 2011). Surprisingly, to the best of our knowledge, no research has focused on the presence of peers when expressing emotions when receiving presents, although it is known that children in general tend to be more expressive when a peer is present (Zeman and Garber, 1996; Shipman et al., 2003; Shahid et al., 2008). Moreover, the presence of an audience, like co-present peers, when receiving reward appears to increase the tendency toward moralistic punishment and one interpretation of this is that an audience may enhance the desire for fairness (Kurzban et al., 2007). Therefore, in the current study, we will take the presence of peers into account when examining emotional expressions after receiving presents.

Secondly, we consider to what extent this effect of peers on children’s expressive behavior interacts with age as a potential factor. Children’s social awareness is known to develop fundamentally between the age of 8 and 11 (Saarni, 1981, 1984). In the Jimmy Kimmel example, the likability of the gift seemed to affect the older sibling’s emotional expressions more than those of the younger boy. Perhaps, the latter did not consider the potato to be the most desirable gift, but he might just have been less aware of his brother’s emotional state than vice versa. In view of theories of developmental differences in social awareness, we may expect older children to be more affected by the presence of a peer than younger ones in their emotional responses (e.g., Piaget, 1950; Saarni, 1984; Ekman, 1992). Indeed, in earlier studies, we found that for 8-year-old children, the social context they found themselves in was of less relevance for the way they expressed their emotions than it was for 11-year-old children (Visser et al., 2014a,b). The current study aims to further explore whether 8-year-old children would express their emotions differently from 11-year-old children, as a function of the event that leads to this emotion (receiving a disappointing or a satisfying present) and the context (in the absence or co-presence of a peer).

Finally, we explore how these emotional expressions may change in the course of a child’s response, where we are specifically interested in the extent to which changes in their assessment of the social context have an impact on the child’s expressive behavior. The Jimmy Kimmel example demonstrated that children’s initial reaction may differ from their later reaction, which appeared to depend on the fact that they became more aware of their peer’s reaction to their Christmas gift. Indeed, emotional expressions are not static experiences, but progress over time (Scherer, 2009). The relative influence of different factors may change in the course of emotional reactions, as people reconsider motives for expressing their emotions in a certain way (Banse and Scherer, 1996; Scherer, 2009). Therefore, we examine how children’s expressions change as a function of how they assess their social context, in particular when they compare their own present with the one another person has just received. We operationalize this by focusing on participants’ expressive behavior before and after they make eye contact with their peer. Before we describe the study in more detail, we first present a short discussion of relevant earlier research.

## Background

A large part of earlier research on emotion has focused on discrete, basic emotions and their universal character (e.g., Tomkins, 1962; Izard, 1971; Ekman, 1992; Darwin, 1998). Discrete emotion theories suggest that children learn to express their emotions through affect programs (Ekman, 1992). These programs are directly linked to the motivational cognitive system and provide people with the ability to experience six prototypical emotions, or a combination of those, which may be accompanied by specific facial expressions (Tomkins, 1962). According to such discrete emotion theories, facial expressions of emotion are considered as universal and similar for all individuals. However, this implication has been questioned by several other (dimensional) approaches on emotions. For example, Russell and Feldman Barrett (1999) started with referring to named emotions (like anger or sadness) as prototypical episodes of core affects (affective feelings), which are not necessarily defined as “basic” or similar to all individuals. According to their theory, emotions are supposed to vary on a continuum of two factors, arousal (passiveness to activeness) and valence (unpleasantness to pleasantness).

Recently, emotion research has been focusing on subjective aspects of emotions, and various studies showed that an individual’s evaluation of a situation may also have an impact on emotional expressions (e.g., Parkinson, 1996; Scherer and Ellgring, 2007; Scherer, 2009; Mumenthaler and Sander, 2012; Fernández-Dols and Crivelli, 2013). According to the componential model of emotions (e.g., Scherer and Ellgring, 2007; Scherer, 2009), emotions are defined as on-going processes in which individuals are continuously estimating and evaluating the significance of situations for their well-being. Various characteristics of the situation may be important for emotion elicitation; for example, the novelty, pleasantness and relevance of the event determine to a large extent the valence and intensity of any emotional response. In this way, emotional expressions are not universal *per se*, but constructed by an individual’s subjective assessment (or *appraisal*) of a situation, which depends on the validation of personal needs, goals and values (e.g., Frijda, 1986; Scherer et al., 2001; Scherer, 2009; Mumenthaler and Sander, 2012). As a result, different people may express the same emotion differently, depending on a variety of appraisals (Mumenthaler and Sander, 2012). Therefore, appraisal theorists claim that emotions are not necessarily static and universal experiences, as these may vary as a function of appraisals (Scherer et al., 2001; Scherer, 2009). In the current experimental set-up, the event of winning the first prize will most likely trigger positive appraisals, and therefore elicit emotions like happiness, while the event of receiving the consolation prize may be expected to trigger more negative appraisals and elicit emotions like disappointment.

Arguably, however, emotional expressions of happiness and disappointment may also be affected by contextual factors, such as the co-presence of a peer. In general, the importance of contextual factors for the construction of emotional expressions has been explained in terms of push and pull effects (e.g., Banse and Scherer, 1996). Push effects of emotions represent how one’s internal state influences the display of emotions. In addition, these expressions need to meet requirements of sociocultural specific models shaped by one’s contextual environment, also known as pull effects. The presence or absence of addressees or spectators, and the interdependence we experience with them in specific situations partly shape this social context (Fridlund, 1991; Parkinson, 1996; Kelley et al., 2003). The concept of pull effects on emotions suggests that people express emotions in the presence of others according to certain social rules that fit the situation they are in (Ekman and Friesen, 1975). These social rules, sometimes referred to as display rules, dictate what kind of expressive behavior is socially appropriate or desirable in certain social contexts and give directions as to how, where, when, and to whom people should express their emotions (Garrett-Peters and Fox, 2007). This implies that the co-presence of peers may affect children’s expressive behavior when receiving disappointing or satisfying presents. Therefore, the first research question we try to answer in this study is formulated as follows:

RQ1: How does the co-presence of a peer influence non-verbal emotional expressions in children when being given a disappointing or satisfying present?

So far, studies have shown that children regulate their emotional expressions to some extent after receiving a *disappointing* present in the presence of *adults* (Saarni, 1984; Cole, 1986; Garner and Power, 1986; Baaken, 2005; Kieras et al., 2005; Garrett-Peters and Fox, 2007; Tobin and Graziano, 2011; Kromm et al., 2015). In experiments applying variations of the so-called mistaken-gift-paradigm, children were asked to rate their desire for a number of toys and books. Next, they were presented with two gift-wrapped boxes in a random order; one box contained their favorite listed item, and the other box contained their least favorite one. Facial expressions in reaction to both presents were videotaped and analyzed. Using this paradigm with children in primary school, studies found that older children smiled more than younger children, even when the present was not the one they desired, whereas younger children’s expressions revealed some level of disappointment when they got the present they desired the least (Saarni, 1984; Garrett-Peters and Fox, 2007).

This can be interpreted as a sign of an increased social awareness, as it shows that older children take into account what is expected from someone who gets a present and use display rules for reacting politely (e.g., by smiling) regardless of whether they appreciate the present or not. Similar studies conducted with younger participants (between the age of three and five) revealed that these children tend to show their disappointment more (Cole, 1986; Garner and Power, 1986; Kieras et al., 2005). Taken together, these results suggest that children gradually learn to regulate their emotional expressions when receiving a disappointing present, which is in line with developmental studies concerning display rules (Saarni, 1981; Gnepp and Hess, 1986; Saarni et al., 2006). According to Gnepp and Hess (1986), a developmental shift across the elementary-school years can be observed, in which children, as they grow older, demonstrate an increased understanding of the appropriateness of specific emotional expressions in specific situations. As children grow older, they are better able to adapt their emotional expressions in order to meet their personal goals and to meet the demands and expectations of their surroundings (Shipman et al., 2003). As we noted above, children’s social awareness and ability to regulate their emotions develops fundamentally between the age of eight and eleven (Saarni, 1981, 1984; Kromm et al., 2015). Around the age of 10, children appear to possess the complex understanding of why certain emotional expressions are appropriate or not in specific situations (Kromm et al., 2015). Indeed, in earlier studies, we found that for 8-year-old children, the social context they found themselves in was of less importance for the way they non-verbally expressed their emotions than it was for 11-year-old children (Visser et al., 2014a,b). Therefore, this study aims to further explore whether children adjust their emotional expressions as a function of the absence or presence of peers and whether this is affected by their age and abilities to regulate their emotional expressions. So, the second research question is formulated as follows:

RQ2: Does age affect children’s expressive behavior in the co-presence of a peer when receiving a disappointing or satisfying present?

Researchers studied the way children respond on disappointing presents using the mistaken-gift-paradigm by focusing on age (Cole, 1986; Garner and Power, 1986; Kieras et al., 2005), culture (Garrett-Peters and Fox, 2007), and strategies children use for regulating their emotions (Zeman and Garber, 1996; Baaken, 2005; Tobin and Graziano, 2011; Kromm et al., 2015). However, to our knowledge, no research so far used a variation of the mistaken-gift-paradigm to study a possible effect of presence of peers. Still, we know that, in general, when people are rewarded for accomplishments, they evaluate and compare their compensations with those of others (e.g., Andreoni et al., 2002). The level of fairness of outcomes tends to trigger more emotional responses than the evaluation of the outcome itself (Barry et al., 2004; Hamilton, 2006). Such reactions appear to be quite instinctive in nature (De Waal, 1997; Brosnan and de Waal, 2003; De Waal and Davis, 2003; Brosnan and de Waal, 2014). De Waal (1997), Brosnan and de Waal (2003), and De Waal and Davis (2003), for example, conducted multiple studies in which capuchin monkeys carried out a task that was rewarded with grapes (food these primates prefer) or pieces of cucumber (food they prefer less than grapes). These monkeys rejected cucumber as a reward once they had been compensated with grapes. Even more relevant for the current research is that they also rejected cucumber once they noticed that other monkeys were being rewarded with grapes. This shows that capuchin monkeys measure reward in relative terms, and they evaluate and compare these reward with those of others. Using a variation of the mistaken gift paradigm, we study whether our child participants act in a similar way.

When children compare their prize with the prize their peer was given, they may adjust the evaluation of their own prize. This implies that emotional reactions, like evaluating individual compensations with those of others, are dynamically adjusted over time, and could vary as a function of changes in appraisals (Scherer, 2009). In other words, events are likely to continuously being re-appraised (Ellsworth and Scherer, 2003). For instance, instinctive initial reactions can evolve into more regulated, socially appropriate secondary reactions. Moreover, although there is support that brief segments of expressive behavior accurately reflect expressive behavior over long durations (Ambady and Rosenthal, 1992), current research suggests that lengthening studied data segments may reveal some sort of second emotional episode in a response, especially in the case of adjusting non-verbal emotional behavior by applying display rules that fit a social context (Garrett-Peters and Fox, 2007). Therefore, it is likely that within the course of receiving an unwanted gift, conflicting appraisals unfold in time (Ellsworth and Scherer, 2003). In this respect, it is interesting to take the role of gaze into account, as it has been argued that the level of social contact is very much influenced by patterns in gaze behavior between people (Argyle and Dean, 1965; Shahid et al., 2012; Borras-Comes et al., 2014). The experience of making eye contact is an important feature for the course of emotional expressions. For example, Shahid et al. (2012) studied how eye contact between children can influence the experience of shared emotions like enjoyment or disappointment. While interacting in a game, children who had direct eye contact with each other showed more enjoyment than children who had no direct eye contact. Therefore, we will not only compare emotional reactions of children who play a game alone and in the presence of a peer, but also compare expressive behavior of the latter before and after they have made eye contact. In this way, we are able to examine how children’s expressions change as a function of how they assess their social context, in particular when they compare their own present with the one another person has just received. Therefore, the third research question is formulated as follows:

RQ3: Do changes in children’s assessments of the social contact affect their expressive behavior in the course of their response when receiving a disappointing or satisfying present?

Taking stock, even though the unwanted gift paradigm has revealed interesting insights into how children respond non-verbally to (un)wanted gifts, to the best of our knowledge no earlier studies have looked into how children respond to wanted and unwanted gifts when they are in the presence of a peer who receives a different (better or worse) gift. This is what we study in the current paper, where in addition, we study whether this non-verbal response is different for younger and older children, and whether there are differences between initial (before eye contact) and secondary (after eye contact) responses.

## Present Study

In the current study, we examined whether the presence of peers affects children’s expressive behavior during the course of a positive or negative event, in particular while receiving a consolation prize (small gift) or a first prize (large gift). In the production experiment, we invited 8- and 11-year-old children to play a game alone (in which they had to compete against the computer) or in pairs (in which they had to compete against each other). The course of the game was manipulated in such a way that it always resulted in a tie, between the child and the computer or between the two children. Subsequently, the experiment leader randomly presented participating children with either the top prize or the consolation prize. In this way, we elicited particular emotional expressions, which we analyzed by conducting two subsequent perception tests, in which we asked third-party judges to rate children’s level of happiness in muted video clips. This research tests whether contextual factors are important for positive and negative emotional expressions (like happiness and disappointment). Due to these factors, people are expected to adjust their emotional expressions with the purpose that someone else will perceive them (Banse and Scherer, 1996). Perception (or judgment) tests are known to be valuable instruments for assessing changes in socially embedded expressive behavior, as the perceptual meaning of expressions is rated by multiple judges (e.g., Kromm et al., 2015). In the first perception test, children’s complete reactions upon receiving their gift were shown to third-party judges. We examined whether these reactions differed depending on whether an opponent was physically present or not for two different age groups. In the second perception test, judges were shown only the reactions of children who had participated in the “in presence of a peer” condition. We split the reactions of participants into two parts, with the moment of mutual eye gaze between the opponents as the cutting point. In this way, we explored how children’s expressive behavior progressed, i.e., before and after they became explicitly aware of their social context.

Lastly, the current studies were carried out in accordance with the recommendations of APA guidelines for conducting experiments, the Netherlands Code of Conduct for Scientific Practice and the Code for Use of Personal Data in Scientific Research (KNAW). The studies were waived by the ethics committee at Tilburg University. All parents gave written consent to the use of their children’s recordings. For the perception studies, all participants gave consent to the use of their data.

## Data Collection

### Method

#### Participants

A total of 86 children participated in this study, of which 41 were 8 years old (*M* = 101.93 months, *SD* = 3.42 months, 27 girls) and 45 were 11 years old (*M* = 137.27 months, *SD* = 3.58 months, 23 girls). Children were randomly assigned to a game condition (competing the computer or a physically present peer) and a reward condition (receiving the consolation prize or the first prize). **Table 1** displays the distribution of child participants across experimental conditions. The experiment was conducted at two primary schools in Zoetermeer, The Netherlands. Beforehand, the parents of the participants were informed about the experimental procedure and asked for their signed permission for their children’s participation and recordings of their performance.

**Table 1:** Distribution of child participants across experimental conditions.

Age	Game context	Consolation prize	First prize	Total for each condition
8-year-olds	Computer	10	9	19
	Present peer	11	11	22
11-year-olds	Computer	11	10	21
	Present peer	12	12	24
			Total of 86 participants		

#### Experimental Procedure

**FIGURE 1:**
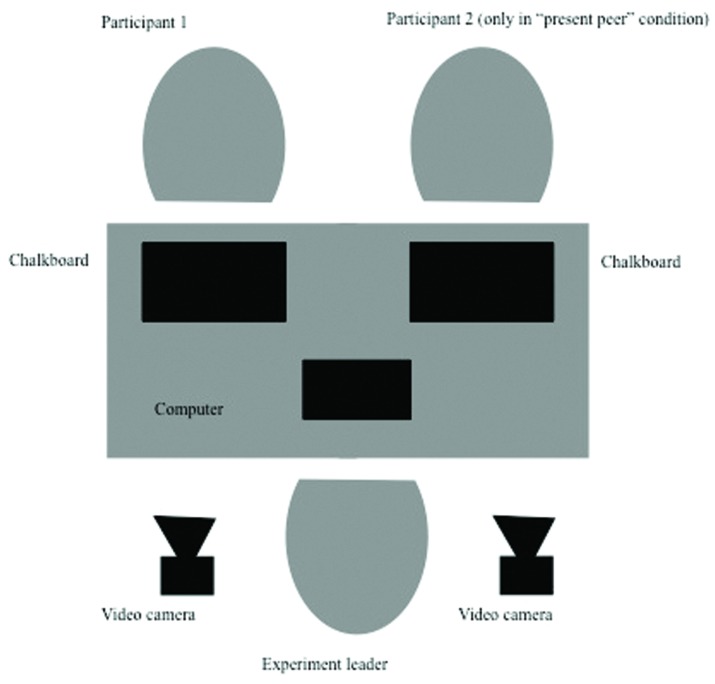
**Experimental setting**.

**FIGURE 2:**
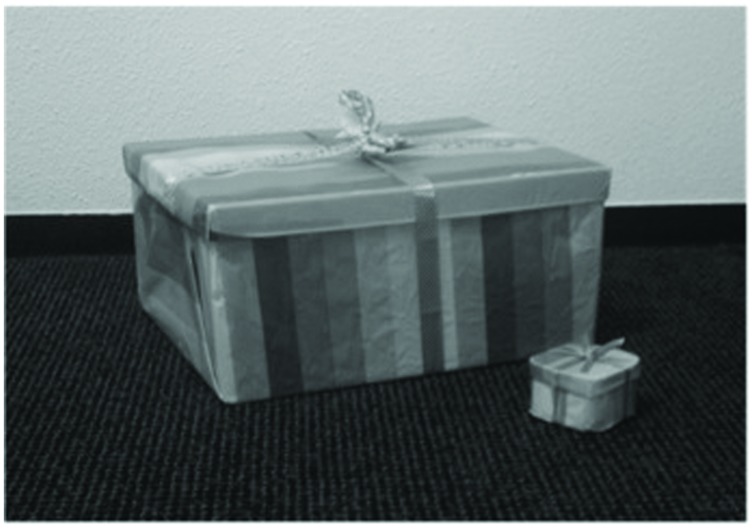
**Representations of first prize and consolation prize (respectively the large and the small package)**.

Children were seated behind a table, facing the experimenter. In the “present peer” condition, they were placed next to each other and were able to see each other’s face and upper body. They were told that they were about to play a game. In the “computer” condition, there was only one child in the experimental room, who had to compete against the computer. Apart from this, the experimental procedures were identical for both conditions. All children were filmed by separate camera’s standing in front of them (see **Figure 1**). The experimenter acted as the game leader, but kept the interaction between her and the children as limited as possible, by leading the game according to a script. She avoided making eye contact with children in both conditions by looking at her computer screen in front of her, which was supposed to be the electronic game board. Before the game, the experimenter explained that the player (either the actual participant in the “present peer” condition, or the virtual participant in the “computer” condition) who would collect most game points would win the first prize, and the other player would receive the consolation prize (again, either the actual participant in the “present peer” condition, or the virtual participant in the “computer” condition). Both gifts were wrapped in paper, so the children could not see what the prizes were. However, the wrapped gifts were shown to them before the game started, and were markedly different, with the first prize being rather big and the consolation prize being considerable smaller (see **Figure 2**). After this introduction, children were asked to indicate how much they would like to win the consolation prize and the first prize, respectively, on a five-point Likert scale, using specific facial representations of the items, a method that is standard in research with children (e.g., Lockl and Schneider, 2002; Visser et al., 2014a,b). Specifically, an unhappy face (corners of the mouth pulled down) represented a score of 1 (“I don’t want this prize at all”), and a happy face (corners of the mouth pulled up) represented a score of 5 (“I want this prize very much”). Children of both age groups had no difficulties in understanding this scale.

Next, children played a guessing game based on the Dutch television show “Wat vindt Nederland?” (English: “What does Holland think?”). Experiments in which children play games is developmentally appropriate for elementary school-aged children. They are familiar with playing structured games and become emotionally aroused easily in game situations because of their emphasis on the importance of winning or losing (Taylor and Asher, 1984). The experimenter presented a number of topics (for example “favorite animal”, or “favorite soda drink”) and asked both players to think of the most likely answer Dutch children of their own age would give (for example, “dolphins” or “Coca Cola”). The children had to write their answer down on a small chalkboard on the table in front of them. Children were told that they were not allowed to give the same answer and the participant who was fastest could remain with their choice. The slowest participant was allowed to come up with a new answer. After the children revealed their answers to the experimenter, she pretended to search in the computer database for the correct answer and assigned one game point to the player whose answer was claimed to be most similar to the answer of most Dutch children. Unbeknownst to the children, this decision was in fact predetermined.

In total, 10 game points were to be divided between the two children (or between the child and the computer, in the “computer” condition). However, the progress of the game was manipulated: each child or pair of children was randomly assigned to one of two scripted game narrations, which always ended in a tie. The course of the game was constructed in such a way that this tie outcome was not revealed before the presentation of the 10th and final concept (in other words, after nine concepts the score was always 4–5). In this way, we tried to maximize engagement for the child participants.

When the game was completed, and had ended in a tie, the experimenter remained acting according to the script, and expressed doubts about what to do in this unexpected situation. After some hesitation, she decided about which gift each child received. In the “present peer” condition, one child received the first prize and the other the consolation prize. In the “computer” condition, children were awarded either the first or the consolation prize, depending on the experimental condition they were in. The experiment leader emphasized that this was a random decision, made intermittent eye contact with the child participants to monitor for understanding and otherwise remained neutral in affect so as not to influence their expressive behavior. Research has shown that the concept of fairness is mainly based on the distribution of gains (Andreoni et al., 2002; Falk et al., 2003). Children gradually learn social rules dictating that expressing negative emotions is unacceptable when losing against a peer who is playing fairly (Hubbard, 2001). By following a script, in which chances of winning were equally distributed for both players through the course of the game, we tried to minimize the risk of emotional expressions of frustration due to a sense of unfairness (although obviously we did expect to encounter expressions of happiness or disappointment). Please note that the experimenter only told children which prize they were awarded with, but did not actually hand it over to them. The children were not given the opportunity to touch or open the present.

Directly following the awarding of the prizes (with a maximum interval of 10 s), the experimenter asked children to indicate how happy they were with their prize, again with the help of the facial representations of a five-point Likert scale. After this, all children were debriefed, and were told they had taken part in an experiment. We asked them if they had noticed anything strange during the game and none of them appeared to be aware of our manipulations. Regardless of the prize they had received after the game, all children were offered a small reward (not dependent on game outcome) for their participation (games and stickers). Each experimental session lasted around 20 min.

### Manipulation Check

Before focusing on how social appraisals affect children’s expressive behavior, we assessed if our game-like experimental paradigm worked as intended. For this, we analyzed children’s self-reported attraction to the first prize and the consolation prize before the game and their self-reported happiness with their gift afterward, using a five-point Likert scale. Naturally, we expected children to indicate a higher desire for the first prize over the consolation prize, and that, accordingly, they would indicate to be happier when they had been given the first prize rather than the consolation one.

We indeed found that children reported a higher desire for the first prize (*M* = 4.90, *SD* = 0.34) than for the consolation prize (*M* = 2.27, *SD* = 1.04), *t*(85) = 21.69, *p* = 0.000. Apparently, all children, regardless of their age or the presence of a peer, wanted to win the first prize over the consolation prize. Moreover, children’s desire scores for both the consolation prize and first prize correlated with the degree of happiness they felt after being appointed with one of the prizes (first prize: *r* = 0.23, *n* = 86, *p* = 0.040; consolation prize: *r* = 0.29, *n* = 86, *p* = 0.010). The more children wanted to have a particular prize, the happier they felt afterward.

An ANOVA with prize, game context and age as factors and indication of happiness afterward as the dependent variable shows that in general, children were happier when being awarded the first prize (*M* = 4.86, *SD* = 0.35) than when being awarded the consolation prize (*M* = 2.95, *SD* = 1.25), *F*(1,84) = 92.41, *p* = 0.000, $\eta_{p}^{2}$ = 0.52. We found no effects of age and game context, age: *F*(1,84) = 0.27, *p* = 0.607; presence: *F*(1,84) = 1.21, *p* = 0.275.

**FIGURE 3:**
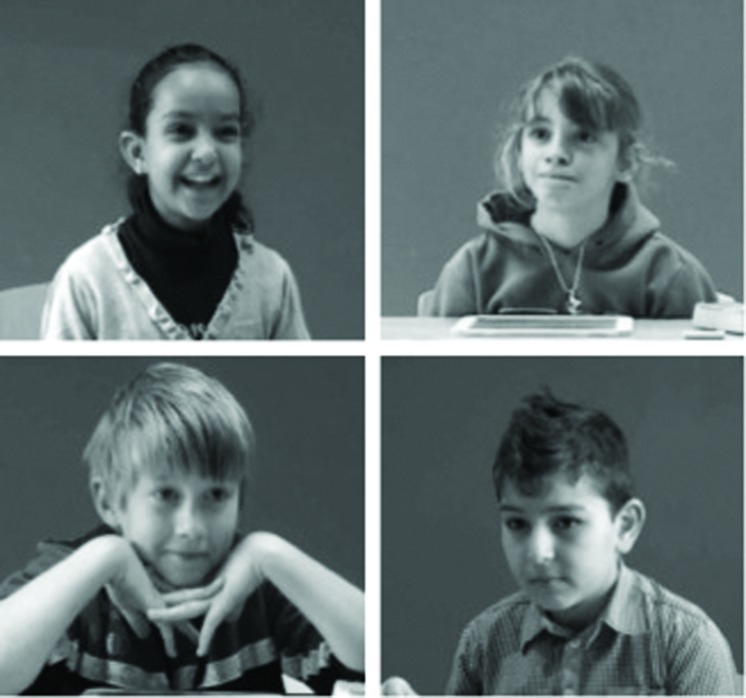
**Stills illustrating representative examples of children’s typical reactions in different experimental conditions (**top left**: computer/first prize; **top right**: present peer/first prize; **bottom left**: computer/consolation prize; **bottom right**: present peer/consolation prize)**.

These results showed that the manipulation worked as intended. Children in all conditions were keener on being awarded the first prize than the consolation prize. Moreover, regardless of their age or of whether they played the game competing the computer or a physically present peer, children reported to be happier with the first price than with the consolation prize. **Figure 3** displays stills from representative reactions of children in all experimental conditions. In the next sections, we analyzed their expressive behavior by letting third-party judges rate children’s level of happiness in two perception experiments.

## Perception Experiment 1 – Complete Fragments

To analyze how children’s expressive behavior is perceived by others, we conducted two perception experiments. In this first perception experiment, we showed third-party judges video clips of complete reactions of children who received either a consolation prize or a first prize.

### Method

#### Participants

In total, 42 adults (24 women), with a mean age of 23 years (*SD* = 6.01) performed as third-party judges in this perception test. All participants were students of Tilburg University who received partial course credits for their participation.

#### Stimuli

For the stimuli in the perception test, we used as many utterances as possible of the ones we recorded in the production experiment, and also made sure that equal numbers of children were selected from the two age groups (8- and 11-year-olds), the two game contexts (competing the computer and competing a physically present peer) and the two game outcomes (first prize and consolation prize). This resulted in a semi-random selection of 72 video fragments of children’s reactions after having been appointed a prize. The selected video fragments were presented to participants and contained children’s reactions to the decision about the distribution of the prizes, from the moment the experimenter determined the winner of the first prize to the moment children were asked to indicate how much they appreciated their prize, with an average length of 8.13 s (*SD* = 2.27). Please note that the fragments did not show children unpacking their prize. The video clips were muted, as the verbal comments of the experimenter announcing who received which gift was likely to influence judgments’ ratings.

#### Procedure

Participants were presented with all 72 video fragments in one of two random orders, to compensate for any order effects due to habituation. Following an identification number (1–72), the actual stimuli were presented one by one. During an inter-stimulus interval of 2.5 s, participants were asked to rate how happy the child appeared to be with the prize it won, on a seven-point Likert scale. To ensure that participants were familiar with the task, the experiment was preceded by a training phase containing four stimuli. Participants completed the perception task individually in a soundproof cubicle.

### Results

A repeated measure ANOVA with prize, game context and age as within-subject factors, and perception of happiness as dependent variable, revealed several main effects and two- and three-way-interactions. Before describing the three-way interaction effect of prize, game context and age on the perceived level of children’s happiness, we will briefly report the main and two-way interaction effects.

First, prize appeared to affect the perception of happiness. As expected, children who won the first prize were perceived to be happier (*M* = 4.73, *SD* = 0.52) than children who won the consolation prize (*M* = 4.22, *SD* = 0.58). Moreover, we found a small main effect of game context on the perception of happiness. Children who played the game in the presence of a peer were perceived happier (*M* = 4.53, *SD* = 0.60) than children who played the game against the computer (*M* = 4.42, *SD* = 0.50). We found no main effect of age. Overall, participants judged 8-year-old and 11-year-old children as equally happy (*M_8-year-olds_* = 4.48, *SD_8-year-olds_* = 0.55; *M_11-year-olds_* = 4.47, *SD_11-year-olds_* = 0.55).

We did find a significant two-way interaction between age and the prize children were presented with on participants’ perception of children’s happiness. A Bonferroni *post hoc* test showed that 8-year-old children were rated as happier when they received the first prize than when they received the consolation prize (*M_firstprize_* = 4.93, *SD_firstprize_* = 0.51; *M_consolationprize_* = 4.04, *SD_consolationprize_* = 0.65). For 11-year-old-children, the type of prize did not affect participants’ perception of their happiness (*M_firstprize_* = 4.54, *SD_firstprize_* = 0.57; *M_consolationprize_* = 4.40, *SD_consolationprize_* = 0.58).

The factor age also interacted with game context on participants’ happiness ratings. *Post hoc* tests (using the Bonferroni method) revealed that when 8-year-old children were playing the game against the computer, they were generally rated as happier than when they were playing against a physically present peer (*M_computer_* = 4.61, *SD_computer_* = 0.50; *M_presentpeer_* = 4.35, *SD_presentpeer_* = 0.68). For 11-year-old children, analyses showed an opposite effect; they were perceived as happier when they played the game together with a physically present peer, than when competing against the computer (*M_computer_* = 4.23, *SD_computer_* = 0.53; *M_presentpeer_* = 4.71, *SD_presentpeer_* = 0.62).

Prize and game context also interacted on the perception of children’s happiness. A Bonferroni *post hoc* test showed that only when receiving the first prize, the physical presence of a peer affected children’s expressions of happiness (*M_computer_* = 4.57, *SD_computer_* = 0.53; *M_presentpeer_* = 4.89, *SD_presentpeer_* = 0.60). When receiving the consolation prize, it did not matter if children were playing against the computer or against a peer, as they were rated as equally (un)happy (*M_computer_* = 4.27, *SD_computer_* = 0.52; *M_presentpeer_* = 4.17, *SD_presentpeer_* = 0.67).

**FIGURE 4:**
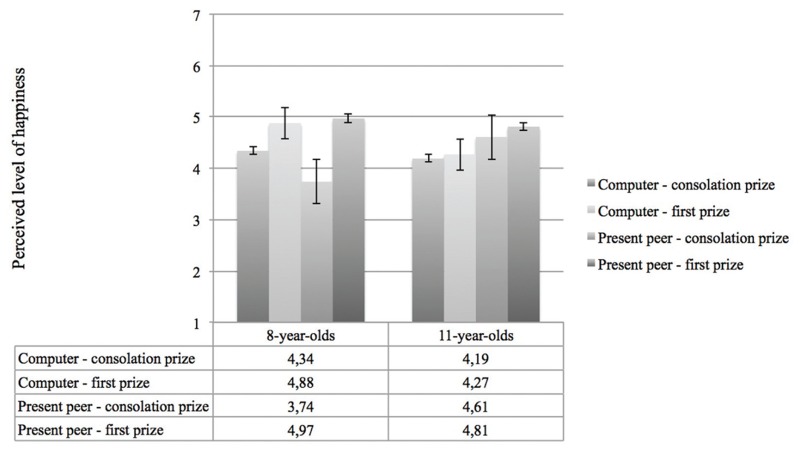
**Perceived level of happiness as a function of age, game context and prize**.

Finally, we found a three-way interaction between prize, game context and age on perceived happiness. **Figure 4** shows that for 8-year-old children, physical presence of a contestant was not important when receiving the first prize; they appeared to be equally happy with it while playing against the computer. However, when 8-year-old children received the consolation price, they seemed to be happier when they played the game against the computer than when they played the game against a peer. In contrast, 11-year-old children who played the game in the “present peer” condition were perceived as happier with both the consolation prize as the first prize. When 11-year-olds played the game competing the computer, they were perceived to be relatively unhappy with both prizes.

All details of the ANOVA analysis can be found in **Table 2**. These results show there is indeed an effect of the co-presence of a peer on how happy children are perceived when being awarded with a disappointing or satisfying prize. In the next section, we zoom in on the emotional expressivity of children who are in the co-presence of a peer and explore how these children’s emotional behavior progressed after receiving a particular present, i.e., before and after they became explicitly aware of their social context (i.e., in the co-presence of a peer). We expected that older children would take the co-presence of a peer even more into account than younger children, as they are more known with appropriate behavior in such situations (Kieras et al., 2005).

**Table 2:** Overview ANOVA’s with perceived level of happiness as independent variable for full fragments.

Factor(s)	*F*	*df*	*p*	*$\eta_{p}^{2}$*
Age	<1	(1, 41)	ns	0.00
Prize	159.83	(1, 41)	0.000	0.80
Game context	7.11	(1, 41)	0.01	0.15
Age * Prize	106.29	(1, 41)	0.000	0.72
Age * Game context	72.82	(1, 41)	0.000	0.64
Prize * Game context	26.95	(1, 41)	0.000	0.40
Age * Prize * Game context	15.13	(1, 41)	0.000	0.27

## Perception Experiment 2 – Split Fragments

Next, we tested the perception by third-party judges of children’s happiness when receiving a prize in different fragments of the child’s reactions. For this, we only used clips from the “present peer” condition, in which we focused on children’s behavior before and after the moment of eye contact between contestants.

### Method

#### Participants

In a second perception task, 42 adults (34 women, *M* = 21.02, *SD* = 2.23) judged a series of video fragments. Again, participants were students of Tilburg University who participated for partial course credit. None of the participants of the second perception task had participated in the first perception task.

#### Stimuli

For this second perception test, we selected all reactions of children who had searched for eye contact with their opponent. The remaining children who where not selected had constantly looked either at the experimenter or simply gazed in front of them. By selecting only children who search for eye contact we were able to precisely define secondary reactions and compare those of both age groups. These reactions were split in two phases; the first phase consisted of children’s initial reaction to their gift before making eye contact with their opponent, the second phase contained their behavior after the moment of eye contact, when they were supposedly more aware of the presence (and gift) of their peer. This resulted in a total amount of 66 video clips, containing initial and secondary reactions of 33 children. All children came from the “present peer” condition, since in the “computer” condition there was no opponent for the participants to make eye contact with. For an overview of the distribution of experimental conditions in the stimuli used in the perception test, see **Table 3**. Similar to the first perception test, stimuli were presented without sounds.

**Table 3:** Selection of stimuli for split fragments perception test.

		Phase before eye contact	Phase after eye contact	Total for each condition
8-year-olds	Consolation prize	8	8	16
	First prize	8	8	16
11-year-olds	Consolation prize	6	6	12
	First prize	11	11	22
			Total of 66 stimuli		

#### Procedure

Since the overall procedure for the second perception test was similar to the procedure of the first perception experiment, we refer to the corresponding section for a more detailed description.

### Results

We analyzed children’s expressions of happiness according to third-party judges by performing a repeated measures ANOVA with age (8- or 11-year-old), prize (consolation prize or first prize) as between-factors and phase of children’s reaction (before or after eye contact) as within-factor. Again, we found a complex three-way interaction effect of age, prize and phase on the perceived level of children’s happiness. In order to understand this interaction better, we will briefly report the main and two-way interaction effects first.

Similar to the results of the first perception test with complete fragments, we found that the type of the prize affected how third-party judges perceived children’s level of happiness. A Bonferroni *post hoc* test showed that children who received the consolation prize were perceived as less happy (*M* = 4.02, *SD* = 0.47) than children who received the first prize (*M* = 4.50, *SD* = 0.41). Moreover, age did not have a main effect on the perceived level of happiness. Again, similar to results of the first perception test, there was an interaction effect of age and the nature of the prize children received on participants’ perception of happiness. A *post hoc* test (Bonferroni method) showed that 8-years-old children were perceived to be happier with the first prize than with the consolation prize (*M*_firstprize_ = 4.58, *SD*_firstprize_ = 0.45; *M*_consolationprize_ = 3.94, *SD*_consolationprize_ = 0.49), whereas 11-year-old children seemed as happy with first prizes as with consolation prizes (*M*_firstprize_ = 4.41, *SD*_firstprize_ = 0.43; *M*_consolationprize_ = 4.11, *SD*_consolationprize_ = 0.51).

Since the aim of this second perception test was to focus on differences in initial an secondary phases of children’s reactions, we were mainly interested in effects including the factor “phase.” First, we found that in general, participants judged children to appear happier in the second phase, so after eye contact (*M* = 4.35, *SD* = 0.45), than in the initial phase, so before eye contact (*M* = 4.17, *SD* = 0.44). Moreover, children’s age interacted with phase on the perception of their happiness. A Bonferroni *post hoc* test revealed that 8-year-old children appeared happier in the initial phase of their reaction than after they had eye contact with their peer (*M_inital_* = 4.46, *SD_initial_* = 0.45; *M_secondary_* = 4.07, *SD_secondary_* = 0.49). However, for 11-year-old children, the opposite was the case; they were initially perceived as less happy, whereas they appeared happier after they had eye contact with their peer (*M_inital_* = 3.88, *SD_inital_* = 0.47; *M_secondary_* = 4.64, *SD_secondary_* = 0.49). There was no interaction between prize and phase. Regardless of eye contact, children were generally perceived happier being awarded the first prize than the consolation prize.

**FIGURE 5:**
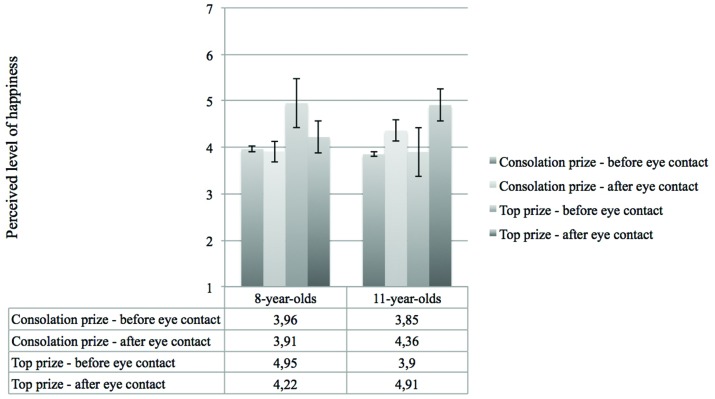
**Perceived level of happiness as a function of age, prize and reaction**.

Finally, we found an interaction between age, prize and phase on the perceived level of happiness. As shown in **Figure 5**, 8-year-old children seemed to be less happy with their first prize as time passed. However, 11-year-old children were perceived to be happier in their reaction after they had eye contact with their opponent, compared to their reaction before they had eye contact, regardless of the type of prize.

Details of the statistical analyses are summarized in **Table 4**.

**Table 4:** Overview ANOVA’s with perceived level of happiness as independent variable for split fragments.

Factor(s)	*F*	*df*	*p*	*$\eta_{p}^{2}$*
Age	<1	(1, 41)	ns	0.00
Prize	158.40	(1, 41)	0.000	0.79
Phase	21.52	(1, 41)	0.000	0.34
Age * Prize	23.37	(1, 41)	0.000	0.36
Age * Phase	249.30	(1, 41)	0.000	0.86
Prize * Phase	1.71	(1, 41)	ns	0.04
Age * Prize * Phase	60.08	(1, 41)	0.000	0.59

## General Discussion and Conclusion

When Jimmy Kimmel asked parents to give their offspring disappointing Christmas presents, this set-up led to interesting reactions of children, which appeared to be in line with what could be predicted based on recent emotional (appraisal) theories that suggest that a variety of social factors are likely to affect emotional expressive behavior (e.g., Manstead and Fischer, 2001; Scherer and Ellgring, 2007; Mumenthaler and Sander, 2012; Fernández-Dols and Crivelli, 2013). The current research systematically investigated how children’s assessments of gifts, the co-presence of a peer and their age may impact their non-verbal expressions of emotion.

The first research question we tried to answer in this study related to how different contextual factors would affect children’s emotional expressions. More specifically, we were interested in how the absence or co-presence of a peer would influence non-verbal emotional expressions in children when being confronted with a disappointing or satisfying event. We found that, in general, children awarded the first prize were perceived as happier than children awarded the consolation prize; similarly, results showed that children who played the game against a physically present contestant were perceived to be happier than children who were playing “alone” against the computer, regardless of the prize they won. Apparently, playing games with a physically present peer was perceived to be more enjoyable than when playing a game alone, which is in line with earlier research (e.g., Shahid et al., 2008). However, to examine how different social appraisals may affect our participants’ emotional reactions, we were specifically interested in any interaction effect of co-presence and prize. Indeed, results showed that when receiving the first prize, children were happier when they were in the presence of a peer who received the consolation prize than when they were alone. On the other hand, when receiving the consolation prize, it did not matter if children were alone or in the presence of a peer, as they were rated as equally (un)happy. Answering our first research question, we can conclude that children’s emotional expressions were indeed affected by contextual factors, albeit only for satisfying events, like being awarded a first prize. However, all children, both those who were playing the game alone and those playing together with a peer, seemed equally disappointed when being awarded the consolation prize. This is in contrast with the results of De Waal (1997), Brosnan and de Waal (2003, 2014), De Waal and Davis (2003); they repeatedly found that primates’ behavior was affected when receiving a disappointing reward, if their peer received a better alternative. An explanation for this may be that these primates lacked certain social skills compared to children, and therefore were less influenced by the social setting than the child participants in our study. However, we need to consider a possible general effect of the experimenter’s presence on the perceived happiness of children in our experiment in the consolation prize condition. It might be that receiving a disappointing present by the experimenter (which can be perceived as rather unfriendly) affected emotional expressions of both children alone and in the co-presence of a peer. Although, we did keep any interaction between the experimenter and the participants as limited as possible (i.e., by following a written script and avoiding any eye contact), due to the nature of the experiment (eliciting spontaneous expressions in a game setting), we were not able to fully control the interaction between the experimenter and the participating children.

The second research question asked whether the concept of age is meaningful in understanding children’s expressive behavior in the co-presence of a peer. As children grow older, they develop certain social skills that may be important for the occurrence of social appraisals for giving meaning to their emotions (Saarni, 1984; Manstead and Fischer, 2001; Saarni et al., 2006; Scherer, 2009). Indeed, when we compared the perceived level of happiness of 8- and 11-year-old children, we found small effects of both prize and co-presence of peers. For 8-year-old children, the physical presence of a contestant was not important when receiving the first prize; they appeared to be equally happy with it. However, when they received the consolation price, they seemed to be happier when they played the game alone than when they played the game together with a peer. This is in line with outcomes of De Waal (1997), Brosnan and de Waal (2003), De Waal and Davis (2003), and Brosnan and de Waal (2014) studying capuchin monkeys. In contrast, 11-year-old children who had played the game with a peer were perceived as happier than 11-year olds who had played against the computer, regardless of which prize they received. When 11-year-olds played the game against the computer, they were perceived to be relatively unhappy with both prizes. These findings supported the view that children gradually learn to adjust their expressive behavior, depending on their social environment. This is in line with studies that used the mistaken gift paradigm, which have shown that age affected children’s reactions while receiving disappointing presents, in a sense that older children showed less disappointment than younger children (Cole, 1986; Garner and Power, 1986; Kieras et al., 2005). However, as we asked judges in our perception tests how *happy* children in presented video clips were; we need to be careful making any assumptions on how *disappointed* children in our study were when receiving the consolation prize. We can only draw conclusions on the perception of their *happiness*. The expression of being less *happy*, as our participants sometimes were perceived as such, may differ from expressions of being *disappointed*, like children studied in the research by Kieras et al. (2005). Therefore, in future research, it would be interesting to study how judges would rate the presence or intensity of other emotions, for example disappointment.

Still, we can conclude that as children grow older, social appraisals get more important and they would show more happiness when receiving a seemingly more disappointing present. So, this study not only provides evidence for an effect of social appraisals when receiving disappointing or satisfying events, but the way children respond emotionally seems to be affected by developmental factors as well.

Finally, we asked how changes in children’s assessments of the social contact, also known as re-appraisals, may affect their expressive behavior in the course of their response. Emotion processes are non-static and dynamically adjusted over time, and have been argued to vary as a function of alternating appraisals (Scherer, 2009). Hence, in the second perception experiment, participants’ expressions were analyzed not only right after they were presented with either the first prize or consolation prize, but also after they had their first post-gift eye contact with their co-present peer. First, we found a small main effect of phase. In general, children were perceived happier after eye contact than before. However, looking at the interaction with age suggests a more nuanced picture. Our findings showed different expressive behavior for both age groups, indicating that eye contact affected the expressive behavior of 8-year-old children in a negative way and that of 11-year-old children in a positive way. The latter seemed happier after they had eye contact with their peer, compared to their initial expression. Similar results were found in a three-way interaction of age, prize and phase. For 11-year-old children, we found no effect of prize and phase for their expressions of happiness, in contrast with 8-year-old children. This again indicated that as children grow older and develop their social skills, their social awareness increases and they adjust their expressive behavior by smiling in the presence of a peer regardless of whether they appreciate their prize or not.

Overall, this research contributes to the idea that emotional expressions are by no means isolated concepts, but are constructed by the evaluation of a (social) context (i.e., social appraisals). Additionally, we have shown that as children’s social awareness increases, their expressions are affected by social appraisals, which may alternate during the course of their response.

## Conflict of Interest Statement

The authors declare that the research was conducted in the absence of any commercial or financial relationships that could be construed as a potential conflict of interest.

## Footnotes

1 The Jimmy Kimmel fragment can be found on YouTube, via the link https://www.youtube.com/watch?v=yWXKUPt7a-U
